# Finite-Time Dissipative Fault Estimate and Event-Triggered Fault-Tolerant Synchronization Control for Discrete Semi-Markov Jumping Neural Networks

**DOI:** 10.3390/e27121186

**Published:** 2025-11-22

**Authors:** Xiaodan Zhu, Yanjun Wang, Yu Chen

**Affiliations:** 1 School of Mathematics and Statistics Science, Ludong University, Yantai 264025, China; 2 School of Chemistry and Chemical Engineering, Ludong University, Yantai 264025, China

**Keywords:** event-triggered, γ-dissipativity, intermediate FE observer, finite-time FTSC, discrete semi-Markov NNs

## Abstract

This paper studies discrete-time semi-Markov neural networks (NNs) and proposes a finite-time γ-dissipative fault estimate (FE) and fault-tolerant synchronization control (FTSC) with the event-triggered method. Based on the intermediate variable introduced, an FE scheme is presented to obtain estimate information of faults, states, and intermediate variables. According to the event-triggered condition designed, an FTSC protocol is proposed so that the error system between master system and slave system is stochasticslly synchronized within finite time and meets γ-dissipativity. An example shows the validity of this proposed scheme.

## 1. Introduction

Neural networks (NNs) that mimic animal NN behavior are an algorithm to process distributed parallel information. Because of the complexity of NNs, the relationships between internal nodes are adjusted to deal with information in network. Regardless of the type of NNs, they have some common characteristics, such as massively parallel processing, distributed storage, and topology elasticity [1,2].

In contrast to pure Markov processes, only the transition time and probability of semi-Markov processes are determined by the time required for the system to reach its present state. Markov theory is named after Russian mathematician Andrei Markov, who pioneered the systematic study of how stochastic processes can be described mathematically. Semi-Markov processes were proposed by Paul Levy in 1954 to build more general models for probabilistic systems. This class of models are used to analyze complex dynamic systems and are often used in reliability calculation. For semi-Markov jump NNs, extended dissipativity was considered by considering partly unknown transition rates [3] or known transition rates [4], and state estimation [5,6] problem are studied by applying different methods, respectively.

Finite-time control [7], due to its characteristic, has been widely applied in various fields. Usually, the system state is required to run within a limited time interval and has good performance indicators within a given time [8,9,10]. For finite-time Markov problems in NNs, the $H_{\infty }$ boundedness [11] was analyzed, synchronization [12] was shown, and $l_{2}$–$l_{\infty }$ state estimation [13] based on unreliable communication links was obtained, respectively. For semi-Markov NN problems, finite-time synchronization control [14,15] was studied, and the $L_{2}$–$L_{\infty }$ synchronization [16] was analyzed, respectively.

Because faults in modern industrial systems are inevitable, faults also happen in NNs. Faults happening may cause serious results. To deal with this problem, fault-tolerant control (FTC) [17] was proposed. The FTC problem [18] in NNs was studied based on ADP methods. The extended FE observer [19] was designed to propose an FTC method for discrete Markovian jump systems. A control protocol with actuator fail-resistant performance was adopted to ensure reliable leader–follower consensus [20]. To save more network resources, a new event-triggering method was proposed which was not constrained by actual system state and designed a reliable observer-based controller [21].

In particular, results [22,23] on semi-Markov NNs existed. Based on the semi-Markov jump model with actuator faults as random variables [22] in NNs, the criterion of passivity FTSC was analyzed. The improved mean-square exponential stability criterion [23] was derived, and then an FTC strategy was proposed by using several decoupling techniques.

Since faults were not considered in [14,15], which motivates our discussion, we discuss a finite-time γ-dissipative FE and event-triggered FTSC for semi-Markov NNs. The highlights of this paper are listed:

(1) The error system between master system and slave system are obtained, and then an intermediate FE method is designed so that the state, fault, and intermediate variables are estimated, which provides information for FTSC protocol proposed. The intermediate observer can obtain better fault estimation results than the general observer [19].
(2) Considering the event-triggered condition designed and estimate information, an FTSC protocol is presented to analyze stochastic FTSC with γ-dissipativity of error systems with finite time. This finite-time scheme can converge quickly and achieve FTSC more quickly than asymptotic stability schemes [19].
(3) The proposed scheme not only estimates faults, but also proposes the FTSC protocol, which belongs to the active FTC category and is more suitable for practical engineering field.

## 2. Problem Statement

A NN as master system is that

(1) $$\begin{array}{ccc} & & x_{m}\left(k+1\right)=-A_{1\varrho (k)}x_{m}\left(k\right)+B_{\varrho (k)}g\left(x_{m}\left(k\right)\right)+C_{\varrho (k)}g\left(x_{m}\left(k-d\left(k\right)\right)\right), \end{array}$$

(2) $$\begin{array}{ccc} & & y_{m}\left(k\right)=A_{2\varrho (k)}x_{m}\left(k\right), \end{array}$$

in which $x_{m}\left(k\right)={\left[x_{m1}\left(k\right),x_{m2}\left(k\right),\ldots ,x_{mn}\left(k\right)\right]}^{T}$ denotes state of neuron’s state; $y_{m}\left(k\right)\in R^{y}$ is measured output; $g\left(x_{m}\left(k\right)\right)=[g_{1}\left(x_{m1}\left(k\right)\right),g_{2}\left(x_{m2}\left(k\right)\right),\ldots ,$$g_{n}\left(x_{mn}\left(l\right)\right){]}^{T}$ denotes neuron activation function; $d\left(k\right)\in R^{d}$ denotes time-varying delay satisfied $0<\underline{d}\leq d\left(k\right)\leq \bar{d}$ and $\underline{d}$, $\bar{d}$ are given; diagonal matrix $A_{1\varrho (k)}>0$; and matrix $B_{\varrho (k)}$, $C_{\varrho (k)}$, $A_{2\varrho (k)}$ are known. For k≥0, ϱ(k) is a discrete-time state semi-Markov process with values in N={1,2,…,N} with $\varrho_{0}$, where $Pr\left\{\varrho \left(k+h\right)=j|\varrho \left(k\right)=i\right\}=r_{ij}\left(h\right)h+o\left(h\right)$ if i≠j, $Pr\left\{\varrho \left(k+h\right)=j|\varrho \left(k\right)=i\right\}=1+r_{ii}\left(h\right)h+o\left(h\right)$ if i=j, and $\lim_{h\to 0}\left(o\left(h\right)/h\right)=0$, $r_{ij}\left(h\right)\geq 0$ is transition rate from mode *i* at *k* to mode *j* at k+h and $r_{ii}\left(h\right)=-\sum_{j\in N,j\neq i}r_{ij}\left(h\right)$. For simplicity, set ϱ(k)=i, then $A_{1\varrho (k)}=A_{1i}>0$, $B_{\varrho (k)}=B_{i}$, $C_{\varrho (k)}=C_{i}$, $A_{2\varrho (k)}=A_{2i}$; the other symbols are similar expression.

A corresponding NN as slave system is that

(3) $$\begin{array}{ccc} & & \;x_{s}\left(k+1\right)=-A_{1i}x_{s}\left(k\right)+B_{i}g\left(x_{s}\left(k\right)\right)+C_{i}g\left(x_{s}\left(k-d\left(k\right)\right)\right)\\ & & \;+D_{i}u_{s}\left(k\right)+E_{i}\omega \left(k\right)+F_{i}f\left(k\right), \end{array}$$

(4) $$\begin{array}{ccc} & & \;y_{s}\left(k\right)=A_{2i}x_{s}\left(k\right), \end{array}$$

in which $x_{s}\left(k\right)$, $g(x_{s}\left(k\right))$, and $y_{s}\left(k\right)$ are similar definitions in master systems, and $u_{s}\left(k\right)\in R^{u}$ denotes the control input. ω(k)∈L[0,∞) denotes external disturbance and $f\left(k\right)\in R^{f}$ denotes the fault, which are different and ω(k) is bounded. $D_{i}$, $E_{i}$, and $F_{i}$ are known matrices.

Define $x\left(k\right)=x_{s}\left(k\right)-x_{m}\left(k\right)$, $y\left(k\right)=y_{s}\left(k\right)-y_{m}\left(k\right)$, then error system between NN (1) and (2) and NN (3) and (4) is

(5) $$\begin{array}{ccc} & & x\left(k+1\right)=-A_{1i}x\left(k\right)+B_{i}g\left(x\left(k\right)\right)+C_{i}g\left(x\left(k-d\left(k\right)\right)\right)\\ & & \;+D_{i}u_{s}\left(k\right)+E_{i}\omega \left(k\right)+F_{i}f\left(k\right), \end{array}$$

(6) $$\begin{array}{ccc} & & y\left(k\right)=A_{2i}x\left(k\right), \end{array}$$

where

$$\begin{array}{ccc} & & g\left(x\left(k\right)\right)=g\left(x_{s}\left(k\right)\right)-g\left(x_{m}\left(k\right)\right),\\ & & g\left(x\left(k-d\left(k\right)\right)\right)=g\left(x_{s}\left(k-d\left(k\right)\right)\right)-g\left(x_{m}\left(k-d\left(k\right)\right)\right). \end{array}$$

**Assumption** **1**  ([24])**.** *In NN (1) and (2) and NN (3) and (4), $g_{l}\left(\cdot \right)$ is bounded and satisfies*

(7) $$\begin{array}{c} {\underline{\varphi }}_{l}\leq \frac{g_{l}\left(x\right)-g_{l}\left(y\right)}{x-y}\leq {\bar{\varphi }}_{l}, \end{array}$$

*where l=1,…,n, y≠x, $g_{l}\left(\cdot \right)=0$, ${\underline{\varphi }}_{l}$${\bar{\varphi }}_{l}$ are known real scalars.*

**Remark** **1.**  *Pointed out in [6], in practice, $r_{ij}\left(h\right)$ is usually partly measurable and bounded with $r_{mij}\leq r_{ij}\left(h\right)\leq r_{Mij}$. In this case, $r_{ij}\left(h\right)$ can be given as follows:*

$$\begin{array}{ccc} & & r_{ij}\left(h\right)=\sum_{k=1}^{K}\phi_{k}r_{ij,k},\\ & & \sum_{k=1}^{K}\phi_{k}=1,\phi_{k}\geq 0, \end{array}$$

*where*

$$\begin{array}{c} r_{ij,k}=\left\{\begin{array}{c} r_{Mij}+\left(k-1\right)\frac{r_{mij}-r_{Mij}}{K-1},i\neq j\\ r_{mij}-\left(k-1\right)\frac{r_{mij}-r_{Mij}}{K-1},i=j \end{array}\right.. \end{array}$$

## 3. State Transformation

Firstly, to better estimate faults, we designed an intermediate observer for system (5) and (6).

An intermediate variable ξ(k) is introduced

(8) $$\begin{array}{c} \xi \left(k\right)=f\left(k\right)-G_{i}x\left(k\right), \end{array}$$

with $G_{i}$ is a gain to be designed. So it is

(9) $$\begin{array}{ccc} & & \;\xi \left(k+1\right)=f\left(k+1\right)+G_{i}A_{1i}x\left(k\right)-G_{i}B_{i}g\left(x\left(k\right)\right)\\ & & \;-G_{i}C_{i}g\left(x\left(k-d\left(k\right)\right)\right)-G_{i}D_{i}u_{s}\left(k\right)\\ & & \;-G_{i}E_{i}\omega \left(k\right)-G_{i}F_{i}G_{i}x\left(k\right)-G_{i}F_{i}\xi \left(k\right). \end{array}$$

For the propose of FE, we designed an intermediate observer:

(10) $$\begin{array}{c} \hat{x}\left(k+1\right)=-A_{1i}\hat{x}\left(k\right)+B_{i}g\left(\hat{x}\left(k\right)\right)\\ \;+C_{i}g\left(\hat{x}\left(k-d\left(k\right)\right)\right)+D_{i}u_{s}\left(k\right)\\ \;+F_{i}\hat{f}\left(k\right)+H_{1i}\left(y\left(k\right)-\hat{y}\left(k\right)\right), \end{array}$$

(11) $$\begin{array}{c} \hat{\xi }\left(k+1\right)=-G_{i}F_{i}\hat{\xi }\left(k\right)-G_{i}D_{i}u_{s}\left(k\right)\\ \;+\left(G_{i}A_{1i}-G_{i}F_{i}G_{i}\right)x\left(k\right)\\ \;+H_{2i}\left(y\left(k\right)-\hat{y}\left(k\right)\right), \end{array}$$

(12) $$\hat{f}\left(k\right)=\hat{\xi }\left(k\right)+G_{i}\hat{x}\left(k\right),$$

(13) $$y\left(k\right)=y\left(k\right)-\hat{y}\left(k\right),$$

where $H_{1i}$, $H_{2i}$ are gains to be designed.

Set $\bar{x}\left(k\right)=x\left(k\right)-\hat{x}\left(k\right)$, $\bar{\xi }\left(k\right)=\xi \left(k\right)-\hat{\xi }\left(k\right)$, $\bar{f}\left(k\right)=f\left(k\right)-\hat{f}\left(k\right)$, we have

(14) $$\begin{array}{ccc} & & \;\bar{x}\left(k+1\right)=\left(-A_{1i}-H_{1i}A_{2i}\right)\left(x\left(k\right)-\hat{x}\left(k\right)\right)+B_{i}\left(g\left(x\left(k\right)\right)-g\left(\hat{x}\left(k\right)\right)\right)\\ & & +C_{i}\left(g\left(x\left(k-d\left(k\right)\right)\right)-g\left(\hat{x}\left(k-d\left(k\right)\right)\right)\right)\\ & & +E_{i}\omega \left(k\right)+F_{i}\left(f\left(k\right)-\hat{f}\left(k\right)\right)\\ & & \;=\left(-A_{1i}-H_{1i}A_{2i}\right)\bar{x}\left(k\right)+B_{i}g\left(\bar{x}\left(k\right)\right)\\ & & \;+C_{i}g\left(\bar{x}\left(k-d\left(k\right)\right)\right)+E_{i}\omega \left(k\right)+F_{i}\bar{f}\left(k\right), \end{array}$$

(15) $$\begin{array}{ccc} & & \;\bar{\xi }\left(k+1\right)=\left(G_{i}A_{1i}-G_{i}F_{i}G_{i}-H_{2i}\right)\left(x\left(k\right)-\hat{x}\left(k\right)\right)-G_{i}B_{i}\left(g\left(x\left(k\right)\right)-g\left(\hat{x}\left(k\right)\right)\right)\\ & & -G_{i}C_{i}\left(g\left(x\left(k-d\left(k\right)\right)\right)-g\left(\hat{x}\left(k-d\left(k\right)\right)\right)\right)-G_{i}E_{i}\omega \left(k\right)\\ & & -G_{i}F_{i}\left(\xi \left(k\right)-\hat{\xi }\left(k\right)\right)+f\left(k+1\right)\\ & & \;=f\left(k+1\right)+\left(G_{i}A_{1i}-G_{i}F_{i}G_{i}-H_{2i}\right)\bar{x}\left(k\right)-G_{i}B_{i}g\left(\bar{x}\left(k\right)\right)\\ & & \;-G_{i}C_{i}(g\left(\bar{x}\left(k-d\left(k\right)\right)\right)-G_{i}E_{i}\omega \left(k\right)-G_{i}F_{i}\bar{\xi }\left(k\right), \end{array}$$

(16) $$\begin{array}{ccc} & & \;\bar{f}\left(k\right)=\left(\xi \left(k\right)-\hat{\xi }\left(k\right)\right)+G_{i}\left(x\left(k\right)-\hat{x}\left(k\right)\right)\\ & & \;=\bar{\xi }\left(k\right)+G_{i}\bar{x}\left(k\right). \end{array}$$

Replacing (16) with (14), it has

(17) $$\begin{array}{ccc} & & \bar{x}\left(k+1\right)=\left(-A_{1i}-H_{1i}A_{2i}+F_{i}G_{i}\right)\bar{x}\left(k\right)+B_{i}g\left(\bar{x}\left(k\right)\right)\\ & & \;+C_{i}g\left(\bar{x}\left(k-d\left(k\right)\right)\right)+E_{i}\omega \left(k\right)+F_{i}\bar{\xi }\left(k\right). \end{array}$$

Let $G_{i}=\rho F_{i}^{T}$ and define $\bar{\bar{x}}\left(k\right)={\left[\begin{array}{cc} {\bar{x}}^{T}\left(k\right) & {\bar{\xi }}^{T}\left(k\right) \end{array}\right]}^{T}$, then

(18) $$\begin{array}{ccc} & & \bar{\bar{x}}\left(k+1\right)=\left({\bar{A}}_{1i}-H_{i}{\bar{A}}_{2i0}\right)\bar{\bar{x}}\left(k\right)+{\bar{B}}_{i}g\left(I_{10}\bar{\bar{x}}\left(k\right)\right)\\ & & \;+{\bar{C}}_{i}g\left(I_{10}\bar{\bar{x}}\left(k-d\left(k\right)\right)\right)+{\bar{E}}_{i}\bar{\omega }\left(k\right), \end{array}$$

where

$$\begin{array}{ccc} & & \;{\bar{A}}_{1i}=\left[\begin{array}{cc} -A_{1i}+\rho F_{i}F_{i}^{T} & F_{i}\\ \rho F_{i}^{T}\left(A_{1i}-\rho F_{i}F_{i}^{T}\right) & -\rho F_{i}^{T}F_{i} \end{array}\right],\\ & & H_{i}=\left[\begin{array}{c} H_{1i}\\ H_{2i} \end{array}\right],I_{10}={\left[\begin{array}{c} I\\ 0 \end{array}\right]}^{T},{\bar{A}}_{2i0}={\left[\begin{array}{c} A_{2i}^{T}\\ 0 \end{array}\right]}^{T},\\ & & {\bar{B}}_{i}=\left[\begin{array}{c} B_{i}\\ -\rho F_{i}^{T}B_{i} \end{array}\right],{\bar{E}}_{i}=\left[\begin{array}{cc} E_{i} & 0\\ -\rho F_{i}^{T}E_{i} & I \end{array}\right],\\ & & {\bar{C}}_{i}=\left[\begin{array}{c} C_{i}\\ -\rho F_{i}^{T}C_{i} \end{array}\right],\bar{\omega }\left(k\right)=\left[\begin{array}{c} \omega_{k}\\ f(k+1) \end{array}\right]. \end{array}$$

**Remark** **2.**  *This paper’s FE design method is an extension of the design method in [25,26,27] to semi-Markov discrete NNs, which provides the basis for the γ-dissipative FTSC.*

**Remark** **3.**  *For the selection of $G_{i}$, scalar ρ is chosen based on experience, and $F_{i}$ is parameter matrix of fault f(k), which restricts the gain of intermediate variable ξ(k) and observer (10)–(13) and improves the estimate ability similarly to the literature [27]. The higher the scalar ρ, the faster the convergence rate, but it also easily cause overshoot phenomenon.*

Next, to reduce the impact of faults, we proposed an event-triggered FTSC protocol.

An event generator is designed [28] to detect and manage the y(k)’s transmission. We set an event-generating function

(19) $$\begin{array}{c} J\left(k\right)={\left(y\left(s_{l}\right)-y\left(k\right)\right)}^{T}\left(y\left(s_{l}\right)-y\left(k\right)\right)-\alpha y^{T}\left(k\right)y\left(k\right), \end{array}$$

in which α∈[0,1), y(k)/$y(s_{l})$ is the current/latest transmitted measurement. When J(k)>0, system signals are transmitted and the transmitted instants set is obtained based on $s_{l+1}=\inf\left\{k\in N|k>s_{l},J\left(k\right)>0,0<s_{0}<s_{1}<\ldots <s_{l}<\ldots \right\}$, where $\left\{s_{0},s_{1},s_{2},\ldots \right\}\subseteq \left\{k_{0},k_{1},k_{2},\ldots \right\}$. Any measurement output variables will be transmitted if J(k)>0. In $\left[s_{l},s_{l+1}\right)$, considering the event-triggered scheme, then

(20) $$\begin{array}{c} {\left(y\left(s_{l}\right)-y\left(k\right)\right)}^{T}\left(y\left(s_{l}\right)-y\left(k\right)\right)\leq \alpha y^{T}\left(k\right)y\left(k\right). \end{array}$$

Based on the above, we designed an FTSC protocol:

(21) $$\begin{array}{c} u_{s}\left(k\right)=D_{i}^{+}S_{c1i}A_{2i}\hat{x}\left(k\right)-D_{i}^{+}S_{c1i}y\left(s_{l}\right)-S_{c2i}\hat{f}\left(k\right), \end{array}$$

where $S_{c1i}$, $S_{c2i}$ are gains to be designed. We selected $S_{c2i}=D_{i}^{+}F_{i}$ with $D_{i}^{+}$ as pseudo-inverse of $D_{i}$, then

(22) $$\begin{array}{ccc} & & u_{s}\left(k\right)=D_{i}^{+}S_{c1i}A_{2i}\hat{x}\left(k\right)-D_{i}^{+}S_{c1i}\bar{y}\left(k\right)\;-D_{i}^{+}S_{c1i}y\left(k\right)-D_{i}^{+}F_{i}\hat{f}\left(k\right), \end{array}$$

in which $\bar{y}\left(k\right)=y\left(k\right)-y\left(s_{l}\right)$, substituting (22) into (5) gives

(23) $$\begin{array}{ccc} & & \;x\left(k+1\right)=A_{1i}x\left(k\right)+B_{i}g\left(x\left(k\right)\right)+C_{i}g\left(x\left(k-d\left(k\right)\right)\right)-S_{c1i}A_{2i}\bar{x}\left(k\right)\\ & & -S_{c1i}\bar{y}\left(k\right)+E_{i}\omega \left(k\right)+F_{i}\bar{\xi }\left(k\right)+F_{i}G_{i}\bar{x}\left(k\right)\\ & & \;=-A_{1i}x\left(k\right)+B_{i}g\left(x\left(k\right)\right)+C_{i}g\left(x\left(k-d\left(k\right)\right)\right)\\ & & +\left({\bar{F}}_{i}-S_{c1i}{\bar{A}}_{2i0}\right)\bar{\bar{x}}\left(k\right)-S_{c1i}\bar{y}\left(k\right)+E_{i0}\bar{\omega }\left(k\right), \end{array}$$

where $\bar{F}=\left[\begin{array}{cc} \rho F_{i}F_{i}^{T} & F_{i} \end{array}\right]$, $E_{i0}=\left[\begin{array}{cc} E_{i} & 0 \end{array}\right]$.

Considering (16), set $\vec{y}\left(k\right)=\left[\begin{array}{c} \bar{x}\left(k\right)\\ \bar{f}\left(k\right) \end{array}\right]$, it can have

(24) $$\begin{array}{c} \vec{y}\left(k\right)={\bar{A}}_{2i}\bar{\bar{x}}\left(k\right). \end{array}$$

For (18) and (24), set $X\left(k\right)=\left[\begin{array}{c} \bar{\bar{x}}\left(k\right)\\ x(k) \end{array}\right]$, $Y\left(k\right)=\left[\begin{array}{c} \vec{y}\left(k\right)\\ x(k) \end{array}\right]$, so it has

(25) $$\begin{array}{ccc} & & \;X\left(k+1\right)={\bar{\bar{A}}}_{1i}X\left(k\right)+{\bar{\bar{B}}}_{i}g(\bar{\bar{I}X\left(k\right))}\;+{\bar{\bar{C}}}_{i}g\left(\bar{\bar{I}}X\left(k-d\left(k\right)\right)\right)\\ & & \;-{\bar{\bar{S}}}_{c1i}\bar{y}\left(k\right)+{\bar{\bar{E}}}_{i}\bar{\omega }\left(k\right), \end{array}$$

(26) $$\begin{array}{ccc} & & \;Y\left(k\right)={\bar{\bar{A}}}_{2i}X\left(k\right), \end{array}$$

where

$$\begin{array}{ccc} & & {\bar{\bar{A}}}_{1i}=\left[\begin{array}{cc} {\bar{A}}_{1i}-H_{i}{\bar{A}}_{2i0} & 0\\ {\bar{F}}_{i}-S_{c1i}{\bar{A}}_{2i0} & -A_{1i} \end{array}\right],{\bar{\bar{B}}}_{i}={\left[\begin{array}{cc} {\bar{B}}_{i}^{T} & B_{i}^{T} \end{array}\right]}^{T},\\ & & {\bar{\bar{C}}}_{i}={\left[\begin{array}{cc} {\bar{C}}_{i}^{T} & C_{i}^{T} \end{array}\right]}^{T},{\bar{\bar{S}}}_{c1i}=\left[\begin{array}{c} 0\\ S_{c1i} \end{array}\right],{\bar{\bar{E}}}_{i}=\left[\begin{array}{c} E_{i}\\ E_{i0} \end{array}\right],\\ & & {\bar{A}}_{2i}=\left[\begin{array}{cc} I & 0\\ \rho F_{i}^{T} & I \end{array}\right],{\bar{\bar{A}}}_{2i}=\left[\begin{array}{cc} {\bar{A}}_{2i} & 0\\ 0 & I \end{array}\right],\bar{\bar{I}}=\left[\begin{array}{cc} I_{10} & I \end{array}\right]. \end{array}$$

For system (25) and (26), definitions and lemmas are used in Section 4 and Section 5.

**Definition** **1**  ([29])**.** *Considering NN (1) and (2) and NN (3) and (4) with given constants $0<\rho_{1}<\rho_{2}$ and matrix R>0, (10)–(13) is an intermediate FE observer to obtain estimates of x(k) and f(k) in system (5) and (6), and (21) is an event-triggered FTSC protocol to compensate the effect of faults so that system (25) and (26) is stochastically synchronized within finite time with $\bar{\omega }\left(k\right)=0$ if*

(27) $$\begin{array}{ccc} & & \;E\left\{X^{T}\left(0\right)RX\left(0\right)\right\}\leq \rho_{1}\;\Rightarrow E\left\{X^{T}\left(k\right)RX\left(k\right)\right\}<\rho_{2}. \end{array}$$

**Definition** **2**  ([30])**.** *For given real matrices $U_{1i}=U_{1i}^{T}$, $U_{2i}$, and $U_{3i}=U_{3i}^{T}$ and constant γ>0, system (25) and (26) is strictly $\left(U_{1i},U_{2i},U_{3i}\right)$-γ-dissipative when X(V(0))=0 if*

(28) $$\begin{array}{ccc} & & \;E\left\{\sum_{k=0}^{S-1}\left[Y^{T}\left(k\right)U_{1i}Y\left(k\right)+2Y^{T}\left(k\right)U_{2i}\bar{\omega }\left(k\right)+{\bar{\omega }}^{T}\left(k\right)U_{3i}\bar{\omega }\left(k\right)\right]\right\}\geq \gamma E\left\{\sum_{k=0}^{S-1}{\bar{\omega }}^{T}\left(k\right)\bar{\omega }\left(k\right)\right\}. \end{array}$$

**Remark** **4.**  *For the strict $\left(U_{1i},U_{2i},U_{3i}\right)$-γ-dissipativity, this performance is equivalent to the other performance if $U_{1i}$, $U_{2i}$, $U_{3i}$ and γ take the particular matrices or constant; that is, setting $U_{1i}=0$, $U_{2i}=I$, $U_{3i}=0$, and γ=0, this performance becomes strict passivity; let $U_{1i}=-I$, $U_{2i}=0$, $U_{3i}={\bar{\gamma }}^{2}I$, γ=0, this performance is standard $H_{\infty }$ performance index $\bar{\gamma }$; when $U_{1i}=I$, $U_{2i}=0$, $U_{3i}=-\beta^{2}I$, γ=0, this performance is the standard $H_{-}$ performance index β. Additionally, the dissipation theory unifies traditional $H_{\infty }$ performance and $H_{-}$ performance.*

**Remark** **5.**  *If system (25) and (26) is strictly $\left(U_{1i},U_{2i},U_{3i}\right)$-γ-dissipative, it must be $\left(U_{1i},U_{2i},U_{3i}\right)$-dissipative. Specifically, system (25) and (26) becomes $\left(U_{1i},U_{2i},U_{3i}\right)$-dissipative if γ=0.*

**Lemma** **1**  ([31])**.** *For any vectors* Ξ, Υ, *and matrix* Θ *with appropriate dimension, the following inequality holds:*

$$\begin{array}{c} 2\Xi^{T}\Upsilon \;\leq \;\Xi^{T}\Theta \Xi +\Upsilon^{T}\Theta^{-1}\Upsilon . \end{array}$$

**Lemma** **2**  ([32])**.** *Considering scalar $\underline{d}$ and matrix $R_{4i}=R_{4i}^{T}>0$ given, the following inequality holds:*

$$\begin{array}{ccc} & & -\underline{d}\sum_{t=k-\underline{d}}^{k-1}\eta^{T}\left(t\right)R_{4i}\eta \left(t\right)\leq -X^{T}\left(k\right)R_{4i}X\left(k\right)+2X^{T}\left(k\right)R_{4i}X\left(k-\underline{d}\right)\\ & & \;-X^{T}\left(k-\underline{d}\right)R_{4i}X\left(k-\underline{d}\right). \end{array}$$

**Lemma** **3**  ([33])**.** *Considering matrices ${\overset{ˇ}{P}}_{j}={\overset{ˇ}{P}}_{j}^{T}>0$, *Π* and scalar κ>0 given, the following inequality holds:*

$$\begin{array}{c} -\Pi^{T}{\overset{ˇ}{P}}_{j}^{-1}\Pi \;\leq \;\kappa^{2}{\overset{ˇ}{P}}_{j}-\kappa \Pi -\kappa \Pi^{T}. \end{array}$$

Subsequently, main result of this paper is shown; i.e., the stochastical finite-time fault-tolerant synchronization with strict $\left(U_{1i},U_{2i},U_{3i}\right)$-γ-dissipativity of system (25) and (26) is analyzed and their existent conditions are shown. Then, gains of the FE and FTSC schemes designed are given.

## 4. γ-Dissipative Finite-Time FTSC Analyse

This section analyse the stochastical FTSC with strict $\left(U_{1i},U_{2i},U_{3i}\right)$-γ-dissipativity of system (25) and (26) within finite time and show the existent condition.

**Theorem** **1.**  *For given scalars γ>0, $0<\rho_{1}<\rho_{2}$, ρ, $\underline{d}$, $\bar{d}$ and matrix R>0, system (25) and (26) achieves stochastical fault-tolerant synchronization within finite time with strict $\left(U_{1i},U_{2i},U_{3i}\right)$-γ-dissipativity if there exist matrices $P_{i}=P_{i}^{T}>0$, $R_{1i}=R_{1i}^{T}>0$, $R_{2i}=R_{2i}^{T}>0$, $R_{3i}=R_{3i}^{T}>0$, $R_{4i}=R_{4i}^{T}>0$, $U_{1i}=U_{1i}^{T}$, $U_{3i}=U_{3i}^{T}$, diagonal matrices $\theta_{1i}>0$, $\theta_{2i}>0$, matrices $T_{12i},T_{13i},T_{23i},T_{24i},T_{3i},U_{2i}$, scalars $\bar{\lambda }$, $\underline{\lambda }$ such that*

(29) $$\begin{array}{ccc} & & \;\left[\begin{array}{ccccccc} \Sigma_{11} & \Sigma_{12} & \Sigma_{13} & \Sigma_{14} & \Sigma_{15} & {\bar{\bar{A}}}_{1i}^{T} & 0\\ {}* & \Sigma_{22} & \Sigma_{23} & 0 & 0 & 0 & \Sigma_{27}\\ {}* & * & \Sigma_{33} & 0 & \Sigma_{35} & \Sigma_{36} & 0\\ {}* & * & * & \Sigma_{44} & \Sigma_{45} & \Sigma_{46} & 0\\ {}* & * & * & * & \Sigma_{55} & 0 & 0\\ {}* & * & * & * & * & \Sigma_{66} & 0\\ {}* & * & * & * & * & * & \Sigma_{77} \end{array}\right]<0, \end{array}$$

(30) $$\begin{array}{ccc} & & \;\underline{\lambda }R-P_{i}<0, \end{array}$$

(31) $$\begin{array}{ccc} & & \;P_{i}-\bar{\lambda }R<0, \end{array}$$

(32) $$\begin{array}{ccc} & & \;\bar{\lambda }\rho_{1}-\rho_{2}\underline{\lambda }<0, \end{array}$$

*where*

$$\begin{array}{ccc} & & \Sigma_{11}=-P_{i}+R_{1i}+R_{2i}-R_{4i}-{\bar{\bar{I}}}^{T}\varphi_{1}\theta_{1i}\bar{\bar{I}}\\ & & \;+\alpha A_{02i}^{T}A_{02i}-{\bar{\bar{A}}}_{2i}^{T}U_{1i}{\bar{\bar{A}}}_{2i},\\ & & \Sigma_{12}=\left[\begin{array}{ccc} R_{4i} & 0 & 0 \end{array}\right],\Sigma_{13}=\left[\begin{array}{cc} {\bar{\bar{I}}}^{T}\frac{\varphi_{2}}{2}\theta_{1i} & 0 \end{array}\right],\\ & & \Sigma_{14}=\left[\begin{array}{cc} 0 & -{\bar{\bar{A}}}_{2i}^{T}U_{2i} \end{array}\right],\Sigma_{15}={\bar{\bar{A}}}_{1i}^{T}T_{3i}-T_{3i},\\ & & \Sigma_{23}=\left[\begin{array}{cc} 0_{31} & \Sigma_{231} \end{array}\right],0_{31}={\left[\begin{array}{ccc} 0 & 0 & 0 \end{array}\right]}^{T},\\ & & \Sigma_{22}=\left[\begin{array}{ccc} \Sigma_{221} & T_{13i}^{T}-T_{23i} & 0\\ {}* & \Sigma_{222} & T_{23i}^{T}-T_{24i}\\ {}* & * & \Sigma_{223} \end{array}\right],\\ & & \Sigma_{27}={\left[\begin{array}{ccc} T_{12i}^{T} & T_{13i}^{T} & 0\\ 0 & T_{23i}^{T} & T_{24i}^{T} \end{array}\right]}^{T},\\ & & \Sigma_{221}=-R_{1i}-R_{4i}+T_{12i}+T_{12i}^{T},\\ & & \Sigma_{223}=-R_{2i}-T_{24i}-T_{24i}^{T},\\ & & \Sigma_{222}=-T_{13i}-T_{13i}^{T}+T_{23i}+T_{23i}^{T}\;-{\bar{\bar{I}}}^{T}\varphi_{1}\theta_{2i}\bar{\bar{I}},\\ & & \Sigma_{231}={\left[\begin{array}{ccc} 0 & {\left({\bar{\bar{I}}}^{T}\frac{\varphi_{2}}{2}\theta_{2i}\right)}^{T} & 0 \end{array}\right]}^{T},\\ & & \Sigma_{33}=diag\left\{-\theta_{1i},-\theta_{2i}\right\},\Sigma_{36}={\left[\begin{array}{cc} {\bar{\bar{B}}}_{i} & {\bar{\bar{C}}}_{i} \end{array}\right]}^{T},\\ & & \Sigma_{35}={\left[\begin{array}{cc} T_{3i}^{T}{\bar{\bar{B}}}_{i} & T_{3i}^{T}{\bar{\bar{C}}}_{i} \end{array}\right]}^{T},\\ & & \Sigma_{44}=diag\left\{-I,-\left(U_{3i}-\gamma I\right)\right\},\\ & & \Sigma_{45}={\left[\begin{array}{cc} -T_{3i}^{T}{\bar{\bar{S}}}_{c1i} & T_{3i}^{T}{\bar{\bar{E}}}_{i} \end{array}\right]}^{T},\\ & & \Sigma_{46}={\left[\begin{array}{cc} -{\bar{\bar{S}}}_{c1i} & {\bar{\bar{E}}}_{i} \end{array}\right]}^{T},\Sigma_{66}=-{\overset{ˇ}{P}}_{j}^{-1},\\ & & \Sigma_{55}=\left(\bar{d}-\underline{d}\right)R_{3i}+{\underline{d}}^{2}R_{4i}-T_{3i}-T_{3i}^{T},\\ & & \Sigma_{77}=diag\left\{-\frac{1}{\bar{d}-\underline{d}}R_{3i},-\frac{1}{\bar{d}-\underline{d}}R_{3i}\right\},\\ & & {\overset{ˇ}{P}}_{j}=\sum_{j=1}^{N}r_{ij}\left(h\right)P_{j},P_{i}=R^{\frac{1}{2}}{\vec{P}}_{i}R^{\frac{1}{2}},\\ & & \underline{\lambda }=\inf\left\{\lambda_{\min}\left({\vec{P}}_{i}\right)\right\},\bar{\lambda }=\sup\left\{\lambda_{\max}\left({\vec{P}}_{i}\right)\right\},\\ & & \varphi_{1}=diag\left\{{\bar{\varphi }}_{1}{\underline{\varphi }}_{1},{\bar{\varphi }}_{2}{\underline{\varphi }}_{2},\ldots ,{\bar{\varphi }}_{n}{\underline{\varphi }}_{n}\right\},\\ & & \varphi_{2}=diag\left\{{\bar{\varphi }}_{1}+{\underline{\varphi }}_{1},{\bar{\varphi }}_{2}+{\underline{\varphi }}_{2},\ldots ,{\bar{\varphi }}_{n}+{\underline{\varphi }}_{n}\right\}. \end{array}$$

**Proof.**  See Appendix A. □

## 5. Gain Design

In this section, the gain design problem of observer (10)–(13) and controller (21) is presented.

**Theorem** **2.**  *For given scalars γ>0, $0<\rho_{1}<\rho_{2}$, ρ, $\underline{d}$, $\bar{d}$ and matrices $R_{1}$, $R_{2}>0$, system (25) and (26) is stochastically fault-tolerant synchronized within finite time and satisfies (28) if there exist matrices $P_{1i}=P_{1i}^{T}>0$, $P_{2i}=P_{2i}^{T}>0$, $R_{11i}=R_{11i}^{T}>0$, $R_{12i}=R_{12i}^{T}>0$, $R_{21i}=R_{21i}^{T}>0$, $R_{22i}=R_{22i}^{T}>0$, $R_{31i}=R_{31i}^{T}>0$, $R_{32i}=R_{32i}^{T}>0$, $R_{41i}=R_{41i}^{T}>0,R_{42i}=R_{42i}^{T}>0$, $U_{11i}=U_{11i}^{T}$, $U_{13i}=U_{13i}^{T}$, $U_{3i}=U_{3i}^{T}$, diagonal matrices $\theta_{1i}>0$, $\theta_{2i}>0$, matrices $U_{12i}$, $T_{121i}$, $T_{122i}$, $T_{123i}$, $T_{124i}$, $T_{131i}$, $T_{132i}$, $T_{133i}$, $T_{134i}$, $T_{231i}$, $T_{232i}$, $T_{233i}$, $T_{234i}$, $T_{241i}$, $T_{242i}$, $T_{243i}$, $T_{244i}$, $T_{31i}$, $T_{32i}$, $U_{21i}$, $U_{22i}$, $H_{i}$, $S_{c1i}$, scalars $\bar{\lambda }$, $\underline{\lambda }$ so that*

(33) $$\begin{array}{ccc} & & \;\left[\begin{array}{cccc} \Xi_{11} & \Xi_{12} & \Xi_{13} & \Xi_{14}\\ {}* & \Xi_{22} & \Xi_{23} & \Xi_{24}\\ {}* & * & \Xi_{33} & \Xi_{34}\\ {}* & * & * & \Xi_{44} \end{array}\right]<0, \end{array}$$

(34) $$\begin{array}{ccc} & & \;\underline{\lambda }diag\left\{R_{1},R_{2}\right\}-diag\left\{P_{1i},P_{2i}\right\}<0, \end{array}$$

(35) $$\begin{array}{ccc} & & \;diag\left\{P_{1i},P_{2i}\right\}-\bar{\lambda }diag\left\{R_{1},R_{2}\right\}<0, \end{array}$$

(36) $$\begin{array}{ccc} & & \;\bar{\lambda }\rho_{1}-\rho_{2}\underline{\lambda }<0, \end{array}$$

*where*

$$\begin{array}{ccc} & & \Xi_{11}=\left[\begin{array}{cc} \Xi_{111} & \Xi_{112}\\ {}* & \Xi_{113} \end{array}\right],\Xi_{22}=\left[\begin{array}{ccc} \Xi_{221} & \Xi_{222} & 0\\ {}* & \Xi_{223} & \Xi_{224}\\ {}* & * & \Xi_{225} \end{array}\right],\\ & & \Xi_{111}=-P_{1i}+R_{11i}+R_{21i}-R_{41i}-I_{10}^{T}\varphi_{1}\theta_{1i}I_{10}-A_{2i}^{T}U_{11i}A_{2i},\\ & & \Xi_{12}=\left[\begin{array}{ccc} \Xi_{121} & 0 & 0 \end{array}\right],\Xi_{112}=-I_{10}^{T}\varphi_{1}\theta_{1i}-A_{2i}^{T}U_{12i},\\ & & \Xi_{113}=-P_{2i}+R_{12i}+R_{22i}-R_{42i}-\varphi_{1}\theta_{1i}-U_{13i}+\alpha A_{2i}^{T}A_{2i},\\ & & \Xi_{221}=\left[\begin{array}{cc} \Xi_{2211} & \Xi_{2212}\\ {}* & \Xi_{2213} \end{array}\right],\Xi_{223}=\left[\begin{array}{cc} \Xi_{2231} & \Xi_{2232}\\ {}* & \Xi_{2233} \end{array}\right],\\ & & \Xi_{222}=\left[\begin{array}{cc} T_{131i}^{T}-T_{121i} & T_{133i}^{T}-T_{122i}\\ T_{132i}^{T}-T_{123i} & T_{134i}^{T}-T_{124i} \end{array}\right],\\ & & \Xi_{2211}=-R_{11i}-R_{41i}+T_{121i}^{T}+T_{121i},\\ & & \Xi_{2213}=-R_{12i}-R_{42i}+T_{124i}^{T}+T_{124i},\\ & & \Xi_{2212}=T_{122i}+T_{123i}^{T},\Xi_{121}=diag\left\{R_{41i},R_{42i}\right\},\\ & & \Xi_{224}=\left[\begin{array}{cc} T_{241i}^{T}-T_{231i} & T_{243i}^{T}-T_{232i}\\ T_{242i}^{T}-T_{233i} & T_{244i}^{T}-T_{234i} \end{array}\right],\\ & & \Xi_{2231}=-T_{131i}-T_{131i}^{T}+T_{231i}+T_{131i}^{T}-I_{10}^{T}\varphi_{1}\theta_{2i}I_{10},\\ & & \Xi_{2232}=-T_{132i}-T_{133i}^{T}+T_{232i}+T_{133i}^{T}-I_{10}^{T}\varphi_{1}\theta_{2i},\\ & & \Xi_{2233}=-T_{134i}-T_{134i}^{T}+T_{234i}+T_{234i}^{T}-\varphi_{1}\theta_{2i},\\ & & \Xi_{225}=\left[\begin{array}{cc} \Xi_{2251} & -T_{242i}-T_{242i}^{T}\\ {}* & -T_{244i}^{T}-T_{244i}-R_{22i} \end{array}\right],\\ & & \Xi_{2251}=-T_{241i}^{T}-T_{241i}-R_{21i},\Xi_{13}=\left[\begin{array}{cccc} \Xi_{131} & 0 & 0 & \Xi_{132} \end{array}\right],\\ & & \Xi_{23}=\left[\begin{array}{cccc} 0 & 0 & \Xi_{231} & 0 \end{array}\right],\Xi_{231}=\left[\begin{array}{ccc} 0 & \Xi_{2311}^{T} & 0 \end{array}\right],\\ & & \Xi_{33}=-diag\left\{\theta_{1i},\theta_{2i},I,\left(U_{3i}-\gamma I\right)\right\},\Xi_{14}=\left[\begin{array}{cccc} \Xi_{141} & X_{142} & 0 & 0 \end{array}\right],\\ & & \Xi_{131}=\left[\begin{array}{c} I_{10}^{T}\frac{1}{2}\varphi_{2}\theta_{1i}\\ \frac{1}{2}\varphi_{2}\theta_{1i} \end{array}\right],\Xi_{132}=\left[\begin{array}{c} -{\bar{A}}_{2i}^{T}U_{21i}\\ -U_{22i} \end{array}\right],\\ & & \Xi_{141}=\left[\begin{array}{cc} \Xi_{1411} & {\bar{F}}_{i}T_{32i}-{\bar{A}}_{2i0}S_{c1i}^{T}\\ 0 & -A_{1i}T_{32i}-T_{32i} \end{array}\right],\\ & & \Xi_{142}=\left[\begin{array}{cc} \Xi_{1421} & {\bar{F}}_{i}T_{32i}-{\bar{A}}_{2i0}S_{c1i}^{T}\\ 0 & -A_{1i}T_{32i} \end{array}\right],\\ & & \Xi_{1411}={\bar{A}}_{1i}^{T}T_{31i}-T_{13i}-{\bar{A}}_{2i0}H_{i}^{T},\Xi_{1421}={\bar{A}}_{1i}^{T}T_{31i}-{\bar{A}}_{2i0}H_{i}^{T},\\ & & \Xi_{24}=\left[\begin{array}{cccc} 0 & 0 & \Xi_{241} & \Xi_{242} \end{array}\right],\Xi_{34}=\left[\begin{array}{cccc} \Xi_{341} & \Xi_{341} & 0 & 0 \end{array}\right],\\ & & \Xi_{341}=\left[\begin{array}{cc} {\bar{B}}_{i}T_{31i} & B_{i}^{T}T_{32i}\\ {\bar{C}}_{i}T_{31i} & C_{i}^{T}T_{32i}\\ 0 & -S_{c1i}^{T}\\ {\bar{E}}_{i}T_{31i} & E_{i0}^{T}T_{32i} \end{array}\right],\Xi_{2311}=\left[\begin{array}{c} \frac{1}{2}I_{10}^{T}\varphi_{2}\theta_{2i}\\ \frac{1}{2}\varphi_{2}\theta_{2i} \end{array}\right],\\ & & \Xi_{241}=\left[\begin{array}{cc} T_{121i} & T_{122i}\\ T_{123i} & T_{124i}\\ T_{131i} & T_{132i}\\ T_{133i} & T_{134i}\\ 0 & 0\\ 0 & 0 \end{array}\right],\Xi_{242}=\left[\begin{array}{cc} 0 & 0\\ 0 & 0\\ T_{231i} & T_{232i}\\ T_{233i} & T_{234i}\\ T_{241i} & T_{242i}\\ T_{243i} & T_{244i}\\ 0 & 0\\ 0 & 0 \end{array}\right],\\ & & \Xi_{44}=diag\left\{\Xi_{441},\Xi_{442},\Xi_{443},\Xi_{444}\right\},\Xi_{443}=\Xi_{444},\\ & & \Xi_{441}=diag\left\{\Xi_{4411},\Xi_{4412}\right\},\Xi_{4422}={\overset{ˇ}{P}}_{2j}-T_{32i}-T_{32i}^{T},\\ & & \Xi_{4411}=\left(\bar{d}-\underline{d}\right)R_{31i}+{\underline{d}}^{2}R_{41i}-T_{31i}-T_{31i}^{T},\\ & & \Xi_{4412}=\left(\bar{d}-\underline{d}\right)R_{32i}+d_{1}^{2}R_{42i}-T_{32i}-T_{32i}^{T},\\ & & \Xi_{442}=diag\left\{\Xi_{4421},\Xi_{4422}\right\},\Xi_{4421}={\overset{ˇ}{P}}_{1j}-T_{31i}-T_{31i}^{T},\\ & & \Xi_{443}=-\frac{1}{\bar{d}-\underline{d}}diag\left\{R_{31i},R_{32i}\right\}. \end{array}$$

*Then the gains $H_{i}$ and $S_{c1i}$ are shown as*

(37) $$\begin{array}{ccc} & & H_{i}=T_{31i}^{-T}H_{i}, \end{array}$$

(38) $$\begin{array}{ccc} & & S_{c1i}=T_{32i}^{-T}S_{c1i}. \end{array}$$

**Proof.**  See Appendix B. □

## 6. An Example

This section presents an example to illustrate the effectiveness and applicability of this finite-time event-triggered γ-dissipative FE and FTSC schemes.

**Figure 1:**
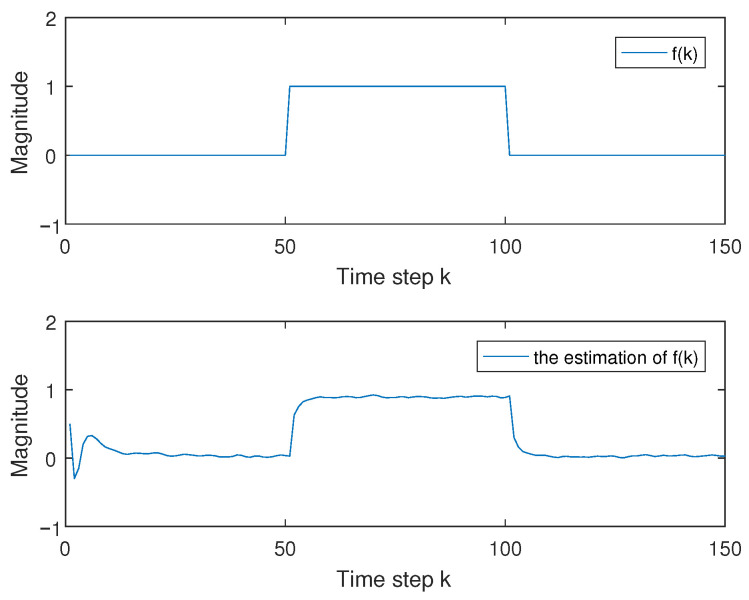
Fault f(k) and its estimation $\hat{f}\left(k\right)$.

**Figure 2:**
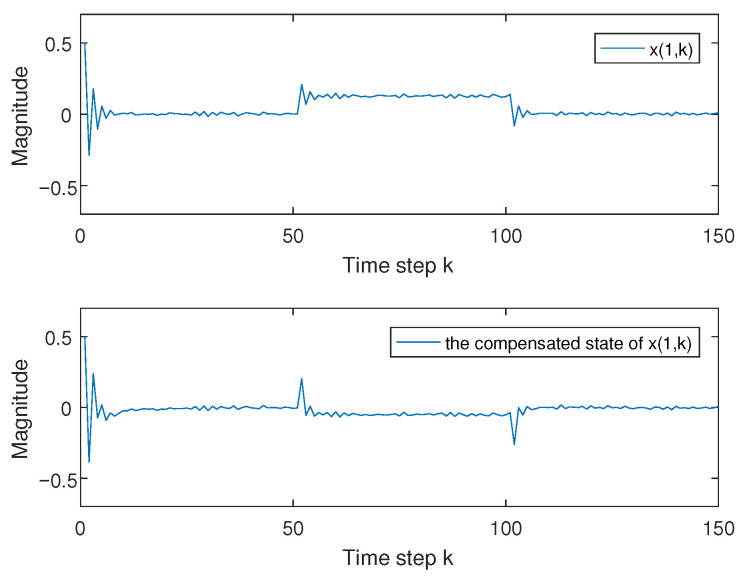
Error state x(1,k) in system (5) and its compensated one.

**Figure 3:**
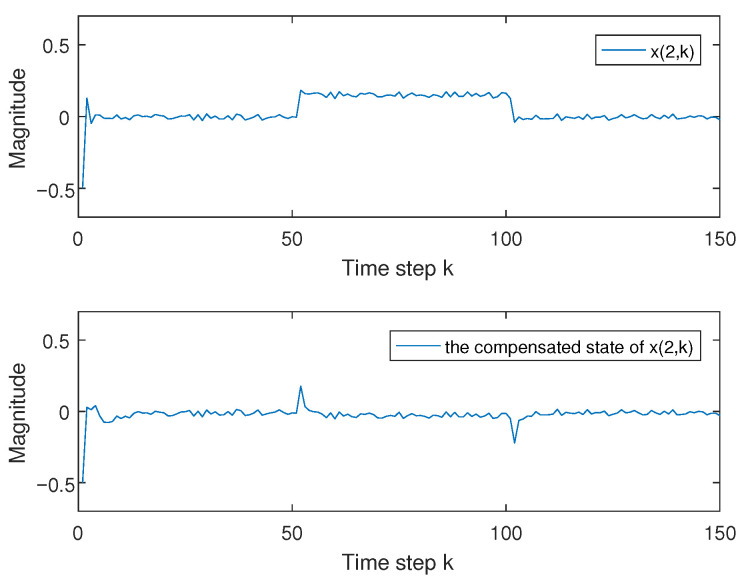
Error state x(2,k) in system (5) and its compensated one.

Assuming that the semi-Markov jump discrete-time NN considered has two modes:

Parameters in Mode 1:

$$\begin{array}{ccc} & & \;A_{11}=\left[\begin{array}{cc} 0.5 & 0\\ 0 & 0.5 \end{array}\right],B_{1}=\left[\begin{array}{cc} -0.1 & 0\\ 0 & 0.2 \end{array}\right],E_{1}=\left[\begin{array}{c} 0.1\\ -0.2 \end{array}\right],\\ & & \;C_{1}=\left[\begin{array}{cc} 0.01 & 0\\ 0 & 0.01 \end{array}\right],D_{1}=\left[\begin{array}{cc} 0.2 & 0\\ 0 & -0.2 \end{array}\right],F_{1}=\left[\begin{array}{c} 0.2\\ 0.2 \end{array}\right],\\ & & \;A_{21}=\left[\begin{array}{cc} 0.1 & 0.1 \end{array}\right], \end{array}$$

and parameters in Mode 2:

$$\begin{array}{ccc} & & \;A_{12}=\left[\begin{array}{cc} 0.2 & 0\\ 0 & 0.5 \end{array}\right],B_{2}=\left[\begin{array}{cc} -0.2 & 0\\ 0 & -0.1 \end{array}\right],E_{2}=\left[\begin{array}{c} 0.1\\ -0.1 \end{array}\right],\\ & & \;C_{2}=\left[\begin{array}{cc} 0.02 & 0\\ 0 & 0.02 \end{array}\right],D_{2}=\left[\begin{array}{cc} -0.3 & 0\\ 0 & 0.4 \end{array}\right],F_{2}=\left[\begin{array}{c} 0.2\\ 0.2 \end{array}\right],\\ & & \;A_{22}=\left[\begin{array}{cc} -0.1 & -0.2 \end{array}\right], \end{array}$$

and $x\left(k\right)=\left[\begin{array}{c} x(1,k)\\ x(2,k) \end{array}\right]$, the activation function [13] is that $g_{a}=\frac{1}{2}\left(\right|x_{a}\left(k\right)+1|-|x_{a}\left(k\right)-1\left|\right)$, a=1,2; it is easy that $\varphi_{1}=diag\left\{0,0\right\}$ and $\varphi_{2}=diag\left\{0.5,0.5\right\}$ by using Assumption 1; $d\left(k\right)=\frac{e^{k}}{e^{k}+1}$ with $\underline{d}=0.5$ and $\bar{d}=1$; and fault f(k) is that $f\left(k\right)=\left\{\begin{array}{c} 1,60<k\leq 120\\ 0,\;\;\;\;\;\;\;\;\;others \end{array}\right..$ The transition rate matrix is chosen as in [6,13,34], which is $-2.2\leq r_{11}\left(h\right)\leq -1.8$, $-1.9\leq r_{22}\left(h\right)\leq -0.5$. We choose the corresponding event-trigger parameter as α=0.7 and K=2 in Remark 1. We consider ρ=−7.5, γ=2, $\rho_{1}=0.9$, $\rho_{2}=2.6$, $R_{1}=I_{3}$, $R_{2}=I_{2}$, and ω(k)=0.2rand−0.1. When $x\left(0\right)=0.5{\left[\begin{array}{cc} 1 & -1 \end{array}\right]}^{T}$, fault f(k) and its estimation $\hat{f}\left(k\right)$ are given in Figure 1, and the error state in (5) between system (1) and system (3) and its compensated error state are given in Figure 2 and Figure 3, which show that controller (19) is effective by the comparison.

Although this example is a numerical examples, it is easy to obtain that this developed scheme is effective through the above analysis and comparison, i.e., semi-Markov NN has some finite-time γ-dissipative FTSC ability based on an event-triggered method in this paper.

**Remark** **6.**  *This paper designs an intermediate observer to estimate faults.*

Based on the utilized information, an event-triggered FTSC protocol is proposed to achieve the FTSC with γ-dissipativity within finite time. The comparison of this scheme with the existing ones are given in Table 1.

By this comparison, it is easy to discover that semi-Markov jump discrete NNs in this paper are more consistent with the actual systems. The practicability of this proposed method is better, which is one of our future works.

## 7. Conclusions

This paper discusses discrete semi-Markov NNs and proposes the finite-time γ-dissipative FE and FTSC by applying an event-triggered theory. Based on the intermediate variable introduced, an FE scheme is designed to estimate the state, faults, and intermediate variables. Based on the estimate information and the event-triggered condition, an FTSC protocol is constructed to obtain the existence conditions of stochastical finite-time fault-tolerant synchronization with the γ-dissipativity of error system. The effectiveness of the proposed solution is proven by an example. In future researches, we will mainly study more appropriate FE and FTC design methods to reduce the conservatism and consider their application in engineering practice, such as robot systems, multi-agent systems, UAV swarms, high-speed train traction systems, and so on.

## Figures and Tables

**Table 1:** The comparison of this scheme with the existing ones.

Reference	Problems	Performance
[6]	Event-triggered $H_{\infty }$ state estimation	$H_{\infty }$ performance
[22]	FTSC	Passivity
[23]	Resilient fault-tolerant anti-synchronization	No
This paper	Finite-time event-triggered FE, FTSC	γ-dissipative

## Data Availability

The original contributions presented in this study are included in the article. Further inquiries can be directed to the corresponding author.

## Appendix A

**Proof of Theorem** **1.** Define a Lyapunov functional:

(A1) $$\begin{array}{ccc} & & \;V\left(X\left(k\right)\right)=X^{T}\left(k\right)P_{i}X\left(k\right)+\sum_{t=k-\underline{d}}^{k-1}X^{T}\left(t\right)R_{1i}X\left(t\right)\\ & & \;+\sum_{t=k-\bar{d}}^{k-1}X^{T}\left(t\right)R_{2i}X\left(t\right)\\ & & \;+\sum_{j=-\bar{d}+1}^{-\underline{d}}\sum_{t=k+j}^{k-1}\eta^{T}\left(t\right)R_{3i}\eta \left(t\right)\\ & & \;+\underline{d}\sum_{j=-\underline{d}}^{-1}\sum_{t=k+j}^{k-1}\eta^{T}\left(t\right)R_{4i}\eta \left(t\right), \end{array}$$

with η(k)=X(k+1)−X(k). When $\bar{\omega }\left(k\right)=0$, it has

(A2) $$\begin{array}{ccc} & & \;E\left\{\Delta V\left(X\left(k\right)\right)\right\}\leq {\left[{\bar{\bar{A}}}_{1i}X\left(k\right)+{\bar{\bar{B}}}_{i}g\left(\bar{\bar{I}}X\left(k\right)\right)+{\bar{\bar{C}}}_{i}g\left(\bar{\bar{I}}X\left(k-d\left(k\right)\right)\right)-{\bar{\bar{S}}}_{c1i}\bar{y}\left(k\right)\right]}^{T}\\ & & \times {\overset{ˇ}{P}}_{j}\left[{\bar{\bar{A}}}_{1i}X\left(k\right)+{\bar{\bar{B}}}_{i}g\left(\bar{\bar{I}}X\left(k\right)\right)+{\bar{\bar{C}}}_{i}g\left(\bar{\bar{I}}X\left(k-d\left(k\right)\right)\right)-{\bar{\bar{S}}}_{c1i}\bar{y}\left(k\right)\right]\\ & & +X^{T}\left(k\right)\left(R_{1i}+R_{2i}\right)X\left(k\right)-X^{T}\left(k-\underline{d}\right)R_{1i}X\left(k-\underline{d}\right)\\ & & -X^{T}\left(k-\bar{d}\right)R_{1i}X\left(k-\bar{d}\right)+\eta^{T}\left(k\right)\left[\left(\bar{d}-\underline{d}\right)R_{3i}+{\underline{d}}^{2}R_{4i}\right]\eta \left(k\right)\\ & & -\sum_{t=k-\bar{d}+1}^{k-\underline{d}}\eta^{T}\left(t\right)R_{3i}\eta \left(t\right)-\underline{d}\sum_{t=k-\underline{d}}^{k-1}\eta^{T}\left(t\right)R_{4i}\eta \left(t\right). \end{array}$$

We set $\zeta \left(k\right)=\left[\begin{array}{c} \zeta_{1}^{T}\left(k\right)\\ {\bar{y}}^{T}\left(k\right)\\ \eta^{T}\left(k\right) \end{array}\right]$, then

(A3) $$\begin{array}{ccc} & & \;2\zeta^{T}T_{1i}\left[X\left(k-\underline{d}\right)-X\left(k-d\left(k\right)\right)\;-\sum_{t=k-d(k)}^{k-\underline{d}-1}\eta \left(t\right)\right]=0, \end{array}$$

(A4) $$\begin{array}{ccc} & & \;2\zeta^{T}T_{2i}[X\left(k-d\left(k\right)\right)-X\left(k-\bar{d}\right)\;-\sum_{t=k-\bar{d}}^{k-d(k)-1}\eta \left(t\right)]=0, \end{array}$$

where

$$\begin{array}{ccc} & & \;\zeta_{1}\left(k\right)=[\begin{array}{cccc} X(k) & X(k-\underline{d}) & X(k-d(k)) & X(k-\bar{d}) \end{array}\\ & & \;\begin{array}{ccc} & g(\bar{\bar{I}}X\left(k\right)) & g(\bar{\bar{I}}X\left(k-d\left(k\right)\right))] \end{array}. \end{array}$$

Based on Lemma 1, one has

(A5) $$\begin{array}{ccc} & & -2\zeta^{T}\left(k\right)T_{1i}\sum_{t=k-d(k)}^{k-\underline{d}-1}\eta \left(t\right)\leq \left(d\left(k\right)-\underline{d}\right)\zeta^{T}\left(k\right)T_{1i}R_{3i}^{-1}T_{1i}^{T}\zeta \left(k\right)\\ & & \;+\sum_{t=k-d(k)}^{k-\underline{d}-1}\eta^{T}\left(t\right)R_{3i}\eta \left(t\right), \end{array}$$

and

(A6) $$\begin{array}{ccc} & & -2\zeta^{T}\left(k\right)T_{2i}\sum_{t=k-\bar{d}}^{k-d(k)-1}\eta \left(t\right)\leq \left(\bar{d}-d\left(k\right)\right)\zeta^{T}\left(k\right)T_{2i}R_{3i}^{-1}T_{2i}^{T}\zeta \left(k\right)\\ & & \;+\sum_{t=k-\bar{d}}^{k-d(k)-1}\eta^{T}\left(t\right)R_{3i}\eta \left(t\right). \end{array}$$

From Lemma 2, it has

(A7) $$\begin{array}{ccc} & & -\underline{d}\sum_{t=k-\underline{d}}^{k-1}\eta^{T}\left(t\right)R_{4i}\eta \left(t\right)\leq -X^{T}\left(k\right)R_{4i}X\left(k\right)+2X^{T}\left(k\right)R_{4i}X\left(k-\underline{d}\right)\\ & & \;-X^{T}\left(k-\underline{d}\right)R_{4i}X\left(k-\underline{d}\right). \end{array}$$

Considering Assumption 1, if diagonal matrix $\theta_{1i}$ exists, one has

(A8) $$\begin{array}{ccc} & & -X^{T}\left(k\right){\bar{\bar{I}}}^{T}\varphi_{1}\theta_{1i}\bar{\bar{I}}X\left(k\right)+X^{T}\left(k\right){\bar{\bar{I}}}^{T}\varphi_{2}\theta_{1i}g\left(\bar{\bar{I}}X\left(k\right)\right)\\ & & -g^{T}\left(\bar{\bar{I}}X\left(k\right)\right)\theta_{1i}g\left(\bar{\bar{I}}X\left(k\right)\right)\geq 0. \end{array}$$

Similarly, it is easy to get

(A9) $$\begin{array}{ccc} & & -X^{T}\left(k-d\left(k\right)\right){\bar{\bar{I}}}^{T}\varphi_{1}\theta_{2i}\bar{\bar{I}}X\left(k-d\left(k\right)\right)\\ & & +2X^{T}\left(k-d\left(k\right)\right){\bar{\bar{I}}}^{T}\frac{1}{2}\varphi_{2}\theta_{2i}g\left(\bar{\bar{I}}X\left(k\right)\right)\\ & & -g^{T}\left(\bar{\bar{I}}X\left(k-d\left(k\right)\right)\right)\theta_{2i}g\left(\bar{\bar{I}}X\left(k-d\left(k\right)\right)\right)\geq 0. \end{array}$$

For

(A10) $$\begin{array}{ccc} & & 2\eta^{T}\left(k\right)T_{3i}^{T}[{\bar{\bar{A}}}_{1i}X\left(k\right)+{\bar{\bar{B}}}_{i}g\left(\bar{\bar{I}}X\left(k\right)\right)+{\bar{\bar{C}}}_{i}g\left(\bar{\bar{I}}X\left(k-d\left(k\right)\right)\right)\\ & & \;-{\bar{\bar{S}}}_{c1i}\bar{y}\left(k\right)-X\left(k\right)-\eta \left(k\right)]=0, \end{array}$$

then

(A11) $$\begin{array}{ccc} & & \;2X^{T}\left(k\right)\left({\bar{\bar{A}}}_{1i}^{T}T_{3i}-T_{3i}\right)\eta \left(k\right)+2g^{T}\left(\bar{\bar{I}}X\left(k\right)\right){\bar{\bar{B}}}_{i}^{T}T_{3i}\eta \left(k\right)\\ & & \;+2g^{T}\left(\bar{\bar{I}}X\left(k-d\left(k\right)\right)\right){\bar{\bar{C}}}_{i}^{T}T_{3i}\eta \left(k\right)-2{\bar{y}}^{T}\left(k\right){\bar{\bar{S}}}_{c1i}^{T}T_{3i}\eta \left(k\right)\\ & & \;-2\eta^{T}\left(k\right)T_{3i}\eta \left(k\right)=0. \end{array}$$

Considering (18), we can have

(A12) $$\begin{array}{ccc} & & \;\alpha y^{T}\left(k\right)y\left(k\right)-{\bar{y}}^{T}\left(k\right)\bar{y}\left(k\right)=\alpha X^{T}\left(k\right)A_{02i}^{T}A_{02i}X\left(k\right)-{\bar{y}}^{T}\left(k\right)\bar{y}\left(k\right)>0, \end{array}$$

with $A_{02i}=\left[\begin{array}{cc} 0 & A_{2i} \end{array}\right]$. Combined with (A1)–(A12), it has

(A13) $$\begin{array}{ccc} & & \;E\left\{\Delta V\left(X\left(k\right)\right)\right\}\leq \zeta^{T}\left(k\right)\bar{\Sigma }\zeta \left(k\right)+\left(\bar{d}-\underline{d}\right)\zeta^{T}\left(k\right)[T_{1i}R_{3i}^{-1}T_{1i}^{T}\\ & & \;+T_{2i}R_{3i}^{-1}T_{2i}^{T}]\zeta \left(k\right)<0, \end{array}$$

in which

$$\begin{array}{ccc} & & \bar{\Sigma }=\left[\begin{array}{ccc} {\bar{\Sigma }}_{1} & {\bar{\Sigma }}_{2} & {\bar{\Sigma }}_{3}\\ {}* & {\bar{\Sigma }}_{4} & {\bar{\Sigma }}_{5}\\ {}* & * & \Sigma_{55} \end{array}\right],{\bar{\Sigma }}_{1}=\left[\begin{array}{ccc} {\bar{\Sigma }}_{11} & \Sigma_{12} & {\bar{\Sigma }}_{13}\\ {}* & \Sigma_{22} & \Sigma_{23}\\ {}* & * & {\bar{\Sigma }}_{33} \end{array}\right],\\ & & {\bar{\Sigma }}_{2}=\left[\begin{array}{c} {\bar{\Sigma }}_{21}\\ 0\\ {\bar{\Sigma }}_{22} \end{array}\right],{\bar{\Sigma }}_{33}=\left[\begin{array}{cc} {\bar{\bar{B}}}_{i}^{T}{\overset{ˇ}{P}}_{j}{\bar{\bar{B}}}_{i}-\theta_{1i} & {\bar{\bar{B}}}_{i}^{T}{\overset{ˇ}{P}}_{j}{\bar{\bar{C}}}_{i}\\ {}* & {\bar{\bar{C}}}_{i}^{T}{\overset{ˇ}{P}}_{j}{\bar{\bar{C}}}_{i}-\theta_{2i} \end{array}\right],\\ & & {\bar{\Sigma }}_{11}={\bar{\bar{A}}}_{1i}^{T}{\overset{ˇ}{P}}_{j}{\bar{\bar{A}}}_{1i}-P_{i}+R_{1i}+R_{2i}-R_{4i}-{\bar{\bar{I}}}^{T}\varphi_{1}\theta_{1i}\bar{\bar{I}}\\ & & \;+\alpha A_{02i}^{T}A_{02i},{\bar{\Sigma }}_{21}=-{\bar{\bar{A}}}_{1i}^{T}{\overset{ˇ}{P}}_{j}{\bar{\bar{S}}}_{c1i},\\ & & {\bar{\Sigma }}_{13}=\left[\begin{array}{cc} {\bar{\bar{I}}}^{T}\frac{\varphi_{2}}{2}\theta_{1i}+{\bar{\bar{A}}}_{1i}^{T}{\overset{ˇ}{P}}_{j}{\bar{\bar{B}}}_{i} & {\bar{\bar{A}}}_{1i}^{T}{\overset{ˇ}{P}}_{j}{\bar{\bar{C}}}_{i} \end{array}\right],\\ & & {\bar{\Sigma }}_{22}=\left[\begin{array}{c} \left(-{\bar{\bar{B}}}_{i}^{T}{\overset{ˇ}{P}}_{j}{\bar{\bar{S}}}_{c1i}\right)\\ \left(-{\bar{\bar{C}}}_{i}^{T}{\overset{ˇ}{P}}_{j}{\bar{\bar{S}}}_{c1i}\right) \end{array}\right],\\ & & {\bar{\Sigma }}_{3}=\left[\begin{array}{ccc} {\left({\bar{\bar{A}}}_{1i}^{T}T_{3i}-T_{3i}\right)}^{T} & 0 & \Sigma_{341} \end{array}\right],\\ & & {\bar{\Sigma }}_{4}={\bar{\bar{S}}}_{c1i}^{T}{\overset{ˇ}{P}}_{j}{\bar{\bar{S}}}_{c1i}-I,{\bar{\Sigma }}_{5}={\bar{\bar{S}}}_{c1i}^{T}T_{3i}-T_{3i}^{T}. \end{array}$$

We set $P_{i}=R^{\frac{1}{2}}{\vec{P}}_{i}R^{\frac{1}{2}}$, then

(A14) $$\begin{array}{ccc} & & \;E\{V(X(k))\}<E\{V(X(0))\}\\ & & \leq E\{X^{T}\left(0\right)P_{i}X\left(0\right)\}\\ & & =E\{X^{T}\left(0\right)R^{\frac{1}{2}}{\vec{P}}_{i}R^{\frac{1}{2}}X\left(0\right)\}\\ & & \leq \sup\lambda_{\max}\left({\vec{P}}_{i}\right)E\left\{X^{T}\left(0\right)RX\left(0\right)\right\}. \end{array}$$

Notice that $E\left\{X_{0}^{T}RX_{0}\right\}<\rho_{1}$, it has

(A15) $$\begin{array}{c} E\left\{V\left(X\left(k\right)\right)\right\}<\sup\lambda_{\max}\left({\vec{P}}_{i}\right)\rho_{1}. \end{array}$$

Based on (30) and (31), we have

(A16) $$\begin{array}{ccc} & & \;E\left\{V\left(X\left(k\right)\right)\right\}>E\left\{X^{T}\left(k\right)P_{i}X\left(k\right)\right\}\\ & & =E\{X^{T}\left(k\right)R^{\frac{1}{2}}{\vec{P}}_{i}R^{\frac{1}{2}}X\left(k\right)\}\\ & & \geq \inf\lambda_{\min}\left({\vec{P}}_{i}\right)E\left\{X^{T}\left(k\right)RX\left(k\right)\right\}. \end{array}$$

Based on (32), it has

(A17) $$\begin{array}{ccc} & & \;E\left\{X^{T}\left(k\right)RX\left(k\right)\right\}<\frac{\sup\lambda_{\max}\left({\vec{P}}_{i}\right)\rho_{1}}{\inf\lambda_{\max}\left({\vec{P}}_{i}\right)}<\frac{\bar{\lambda }\rho_{1}}{\underline{\lambda }}<\rho_{2}. \end{array}$$

So system (25) without $\bar{\omega }\left(k\right)$ is stochastically fault-tolerant synchronized within finite time. In the following, we analyse the strict $\left(U_{1i},U_{2i},U_{3i}\right)$-γ-dissipativity of system (25) and (26) with $\bar{\omega }\neq 0$. We define

(A18) $$\begin{array}{ccc} & & J\left(k\right)=E\{\Delta V\left(X\left(k\right)\right)-[Y^{T}\left(k\right)U_{1i}Y\left(k\right)+2Y^{T}\left(k\right)U_{2i}\bar{\omega }\left(k\right)\\ & & \;+{\bar{\omega }}^{T}\left(k\right)\left(U_{3i}-\gamma I\right)\bar{\omega }\left(k\right)]\}, \end{array}$$

then

(A19) $$\begin{array}{ccc} & & \;J\left(k\right)\leq [{\bar{\bar{A}}}_{1i}X\left(k\right)+{\bar{\bar{B}}}_{i}g\left(\bar{\bar{I}}X\left(k\right)\right)+{\bar{\bar{C}}}_{i}g\left(\bar{\bar{I}}X\left(k-d\left(k\right)\right)\right)\\ & & -{\bar{\bar{S}}}_{c1i}\bar{y}\left(k\right)+E_{i}\bar{\omega }\left(k\right){]}^{T}{\overset{ˇ}{P}}_{j}[{\bar{\bar{A}}}_{1i}X\left(k\right)+{\bar{\bar{B}}}_{i}g\left(\bar{\bar{I}}X\left(k\right)\right)\\ & & +{\bar{\bar{C}}}_{i}g\left(\bar{\bar{I}}X\left(k-d\left(k\right)\right)\right)-{\bar{\bar{S}}}_{c1i}\bar{y}\left(k\right)+E_{i}\bar{\omega }\left(k\right)]\\ & & +X^{T}\left(k\right)\left(R_{1i}+R2i\right)X^{T}\left(k\right)\\ & & -X^{T}\left(k-\underline{d}\right)R_{1i}X\left(k-\underline{d}\right)\\ & & -X^{T}\left(k-\bar{d}\right)R_{1i}X\left(k-\bar{d}\right)\\ & & +\eta^{T}\left(k\right)\left[\bar{d}-\underline{d}\right)R_{3i}+d_{1}^{2}R_{4i}]\eta \left(k\right)\\ & & -\sum_{t=k-\bar{d}+1}^{k-\underline{d}}\eta^{T}\left(t\right)R_{3i}\eta \left(t\right)\\ & & -\underline{d}\sum_{t=k-\underline{d}}^{k-1}\eta^{T}\left(t\right)R_{4i}\eta \left(t\right)\;-[Y^{T}\left(k\right)U_{1i}Y\left(k\right)\\ & & +2Y^{T}\left(k\right)U_{2i}\bar{\omega }\left(k\right)+{\bar{\omega }}^{T}\left(k\right)\left(U_{3i}-\gamma I\right)\bar{\omega }\left(k\right)]. \end{array}$$

When $\bar{\omega }\left(k\right)\neq 0$, it has

(A20) $$\begin{array}{ccc} & & \;2X^{T}\left(k\right)\left({\bar{\bar{A}}}_{1i}^{T}T_{3i}-T_{3i}\right)\eta \left(k\right)+2g^{T}\left(\bar{\bar{I}}X\left(k\right)\right){\bar{\bar{B}}}_{i}^{T}T_{3i}\eta \left(k\right)\\ & & \;+2g^{T}\left(\bar{\bar{I}}X\left(k-d\left(k\right)\right)\right){\bar{\bar{C}}}_{i}^{T}T_{3i}\eta \left(k\right)-2{\bar{y}}^{T}\left(k\right){\bar{\bar{S}}}_{c1i}^{T}T_{3i}\eta \left(k\right)\\ & & \;+2{\bar{\omega }}^{T}{\bar{\bar{E}}}_{i}^{T}T_{3i}\eta \left(k\right)+2{\bar{\omega }}^{T}{\bar{\bar{E}}}_{i}^{T}T_{3i}\eta \left(k\right)-2\eta^{T}\left(k\right)T_{3i}\eta \left(k\right)=0. \end{array}$$

Based on (A3)–(A9) and (A20), we have

(A21) $$\begin{array}{ccc} & & \;J\left(k\right)\leq \zeta^{T}\left(k\right)\bar{\Sigma }\zeta \left(k\right)+\left(d_{2}-d_{1}\right)\zeta^{T}\left(k\right)[T_{1i}R_{3i}^{-1}T_{1i}^{T}\\ & & +T_{2i}R_{3i}^{-1}T_{2i}^{T}]\zeta \left(k\right)+2X^{T}\left(k\right)A_{1i}^{T}{\overset{ˇ}{P}}_{j}{\bar{\bar{E}}}_{i}\bar{\omega }\left(k\right)\\ & & +2{\bar{y}}^{T}{\bar{S}}_{c1i}^{T}{\overset{ˇ}{P}}_{j}{\bar{\bar{E}}}_{i}\bar{\omega }\left(k\right)+2g^{T}\left(\bar{\bar{I}}X\left(k\right)\right)B_{i}^{T}{\overset{ˇ}{P}}_{j}{\bar{\bar{E}}}_{i}\bar{\omega }\left(k\right)\\ & & +2g^{T}\left(\bar{\bar{I}}X\left(k-d\left(k\right)\right)\right)B_{i}^{T}{\overset{ˇ}{P}}_{j}{\bar{\bar{E}}}_{i}\bar{\omega }\left(k\right)\\ & & +2{\bar{\omega }}^{T}{\bar{\bar{E}}}_{i}^{T}T_{3i}\eta \left(k\right)+{\bar{\omega }}^{T}\left(k\right){\bar{\bar{E}}}_{i}^{T}{\overset{ˇ}{P}}_{j}{\bar{\bar{E}}}_{i}\bar{\omega }\left(k\right)\\ & & -[Y^{T}\left(k\right)U_{1i}Y\left(k\right)+2Y^{T}\left(k\right)U_{2i}\bar{\omega }\left(k\right)\\ & & +{\bar{\omega }}^{T}\left(k\right)\left(U_{3i}-\gamma I\right)\bar{\omega }\left(k\right)]. \end{array}$$

Considering (29), J(k)<0 holds. Thus

(A22) $$\begin{array}{ccc} & & \;E\{\Delta V\left(X\left(k\right)\right)<[Y^{T}\left(k\right)U_{1i}Y\left(k\right)+2Y^{T}\left(k\right)U_{2i}\bar{\omega }\left(k\right)\\ & & \;+{\bar{\omega }}^{T}\left(k\right)\left(U_{3i}-\gamma I\right)\bar{\omega }\left(k\right)]. \end{array}$$

Summing both side of (A22) from 0 to S−1, then

(A23) $$\begin{array}{ccc} & & E\left\{V\left(X\left(k\right)\right)\right\}<E\{\sum_{k=0}^{S-1}[Y^{T}\left(k\right)U_{1i}Y\left(k\right)+2Y^{T}\left(k\right)U_{2i}\bar{\omega }\left(k\right)\\ & & \;+{\bar{\omega }}^{T}\left(k\right)\left(U_{3i}-\gamma I\right)\bar{\omega }\left(k\right)]\}+E\left\{V\left(X\left(0\right)\right)\right\}. \end{array}$$

Hence, system (25) and (26) is strictly $\left(U_{1i},U_{2i},U_{3i}\right)$-γ-dissipative. That finishes the proof. □

## Appendix B

**Proof of Theorem** **2.** Because $-P_{j}^{-1}$ in (29) is nonlinear, we multiply left by $T_{3i}^{T}$ and right by $T_{3i}$ to (29), $-T_{3i}^{T}{\overset{ˇ}{P}}_{j}^{-1}T_{3i}<{\overset{ˇ}{P}}_{j}-T_{3i}-T_{3i}^{T}$ holds based on Lemma 3, where $T_{3i}=diag\left\{I,I,I,I,I,T_{3i},I\right\}$. So it has

(A24) $$\begin{array}{c} \left[\begin{array}{ccccccc} \Sigma_{11} & \Sigma_{12} & \Sigma_{13} & \Sigma_{14} & \Sigma_{15} & {\tilde{\Sigma }}_{16} & 0\\ {}* & \Sigma_{22} & \Sigma_{23} & 0 & 0 & 0 & \Sigma_{27}\\ {}* & * & \Sigma_{33} & \Sigma_{34} & \Sigma_{35} & {\tilde{\Sigma }}_{36} & 0\\ {}* & * & * & \Sigma_{44} & \Sigma_{45} & {\tilde{\Sigma }}_{46} & 0\\ {}* & * & * & * & \Sigma_{55} & 0 & 0\\ {}* & * & * & * & * & {\tilde{\Sigma }}_{66} & 0\\ {}* & * & * & * & * & * & \Sigma_{77} \end{array}\right]<0, \end{array}$$

where

$$\begin{array}{ccc} & & {\tilde{\Sigma }}_{16}={\bar{\bar{A}}}_{1i}^{T}T_{3i},{\tilde{\Sigma }}_{46}={\left[\begin{array}{cc} -T_{3i}^{T}{\bar{\bar{S}}}_{c1i} & T_{3i}^{T}{\bar{\bar{E}}}_{i} \end{array}\right]}^{T},\\ & & {\tilde{\Sigma }}_{36}={\left[\begin{array}{cc} T_{3i}^{T}{\bar{\bar{B}}}_{i} & T_{3i}^{T}{\bar{\bar{C}}}_{i} \end{array}\right]}^{T},{\tilde{\Sigma }}_{66}={\overset{ˇ}{P}}_{j}-T_{3i}-T_{3i}^{T}. \end{array}$$

We define

$$\begin{array}{ccc} & & \;P_{i}=diag\left\{P_{1i},P_{2i}\right\},R=diag\left\{R_{1},R_{2}\right\},R_{1i}=diag\left\{R_{11i},R_{12i}\right\},\\ & & \;R_{2i}=diag\left\{R_{21i},R_{22i}\right\},R_{3i}=diag\left\{R_{31i},R_{32i}\right\},R_{4i}=diag\left\{R_{41i},R_{42i}\right\}, \end{array}$$

$$\begin{array}{ccc} & & \;U_{1i}=\left[\begin{array}{cc} U_{11i} & U_{12i}\\ {}* & U_{13i} \end{array}\right],T_{12i}=\left[\begin{array}{cc} T_{121i} & T_{122i}\\ T_{123i} & T_{124i} \end{array}\right],T_{3i}=\left[\begin{array}{cc} T_{31i} & 0\\ 0 & T_{32i} \end{array}\right],U_{2i}=\left[\begin{array}{c} U_{21i}\\ U_{22i} \end{array}\right],\\ & & \;T_{13i}=\left[\begin{array}{cc} T_{131i} & T_{132i}\\ T_{133i} & T_{134i} \end{array}\right],T_{23i}=\left[\begin{array}{cc} T_{231i} & T_{232i}\\ T_{233i} & T_{234i} \end{array}\right],T_{24i}=\left[\begin{array}{cc} T_{241i} & T_{242i}\\ T_{243i} & T_{244i} \end{array}\right], \end{array}$$

and set $H_{i}^{T}=H_{i}^{T}T_{31i}$, $S_{c1i}^{T}=S_{c1i}^{T}T_{32i}$, then (33)–(38) hold based on (29)–(32). That finishes the proof. □
